# Diurnal cycling of rhizosphere bacterial communities is associated with shifts in carbon metabolism

**DOI:** 10.1186/s40168-017-0287-1

**Published:** 2017-06-24

**Authors:** Christopher Staley, Abigail P. Ferrieri, Malak M. Tfaily, Yaya Cui, Rosalie K. Chu, Ping Wang, Jared B. Shaw, Charles K. Ansong, Heather Brewer, Angela D. Norbeck, Meng Markillie, Fernanda do Amaral, Thalita Tuleski, Tomás Pellizzaro, Beverly Agtuca, Richard Ferrieri, Susannah G. Tringe, Ljiljana Paša-Tolić, Gary Stacey, Michael J. Sadowsky

**Affiliations:** 1 BioTechnology Institute, University of Minnesota, 140 Gortner Lab, 1479 Gortner Ave, Saint Paul, MN 55108 USA; 2 Environmental Molecular Sciences Laboratory, Pacific Northwest National Laboratory, Richland, WA 99354 USA; 3 Division of Plant Science and Biochemistry, C.S. Bond Life Science Center, University of Missouri, Columbia, MO 65211 USA; 4 Biological Sciences Division, Pacific Northwest National Laboratory, Richland, WA 99354 USA; 5 Department of Chemistry, University of Missouri Research Reactor, Columbia, MO 65211 USA; 6 Microbial Systems Group, Metagenome Program, DOE Joint Genome Institute, Walnut Creek, CA 94598 USA

**Keywords:** Bacterial community structure, Diurnal rhythm, Rhizosphere, *Arabidopsis*

## Abstract

**Background:**

The circadian clock regulates plant metabolic functions and is an important component in plant health and productivity. Rhizosphere bacteria play critical roles in plant growth, health, and development and are shaped primarily by soil communities. Using Illumina next-generation sequencing and high-resolution mass spectrometry, we characterized bacterial communities of wild-type (Col-0) *Arabidopsis thaliana* and an acyclic line (OX34) ectopically expressing the circadian clock-associated *cca*1 transcription factor, relative to a soil control, to determine how cycling dynamics affected the microbial community. Microbial communities associated with *Brachypodium distachyon* (BD21) were also evaluated.

**Results:**

Significantly different bacterial community structures (*P =* 0.031) were observed in the rhizosphere of wild-type plants between light and dark cycle samples. Furthermore, 13% of the community showed cycling, with abundances of several families, including *Burkholderiaceae*, *Rhodospirillaceae*, *Planctomycetaceae*, and *Gaiellaceae*, exhibiting fluctuation in abundances relative to the light cycle. However, limited-to-no cycling was observed in the acyclic CCAox34 line or in soil controls. Significant cycling was also observed, to a lesser extent, in *Brachypodium*. Functional gene inference revealed that genes involved in carbohydrate metabolism were likely more abundant in near-dawn, dark samples. Additionally, the composition of organic matter in the rhizosphere showed a significant variation between dark and light cycles.

**Conclusions:**

The results of this study suggest that the rhizosphere bacterial community is regulated, to some extent, by the circadian clock and is likely influenced by, and exerts influences, on plant metabolism and productivity. The timing of bacterial cycling in relation to that of *Arabidopsis* further suggests that diurnal dynamics influence plant-microbe carbon metabolism and exchange. Equally important, our results suggest that previous studies done without relevance to time of day may need to be reevaluated with regard to the impact of diurnal cycles on the rhizosphere microbial community.

**Electronic supplementary material:**

The online version of this article (doi:10.1186/s40168-017-0287-1) contains supplementary material, which is available to authorized users.

## Background

The plant rhizosphere contains a complex microbial community that directly impacts plant growth, health, and development [1]. The rhizosphere bacterial community composition is predominantly determined by the soil community, and associated environmental factors (e.g., climate), but is also influenced to some extent by plant host genotype [2, 3]. Relative to bulk soil, a “rhizosphere effect” results in a plant-root-associated microbial community that is more numerous and shows greater metabolic activity that is shaped by exudation of carbon and other metabolites from the plant root [4]. Thus, mutations in genes related to plant nutrient metabolism may in turn influence the rhizosphere microbial community, with implications for plant growth and health [5]. Furthermore, manipulation of host-associated microbial communities is receiving increasing attention as a biological mechanism to improve plant growth and stress resistance [6].

Important to the determination of what constitutes a beneficial microbial community is the characterization of how diverse soil bacteria interact with host plant species [7]. The rhizosphere effect that exerts some control on the rhizosphere community composition is mediated by metabolic exchange between the roots and soil [4]. The partitioning of nutrients to the roots and their exchange with the soil environment is controlled by the response of plants to environmental signals, such as light and temperature [8]. These responses are modulated by a plant’s innate ability to estimate time within an approximately 24-h period and synchronize biological events via the circadian clock [8]. The importance of light in shaping the outcome of host-microbe interactions is becoming increasingly evident. Recent studies show that the intestinal microbiota of humans [9] and mice [10, 11] undergo diurnal oscillations under the control of host feeding time and diet, and silencing of a host’s molecular clock genes causes gut dysbiosis [12].

The circadian clock is an important regulator of numerous basic plant functions including central carbon metabolism [13], gene expression, stomatal function, and the timing component of photoperiodism, which regulates seasonal reproduction [14, 15]. The clock is also subject to extensive natural variation both within and between species, and this is reported to influence plant fitness and performance [16–20]. As a result, the circadian clock is considered a key regulator of plant physiology and adaptation to different geographic environments, enabling an organism to anticipate periodic environmental changes and adapt its physiological and developmental states accordingly [8, 21]. Indeed, the life cycles of pathogens are closely associated with diurnally regulated host plant metabolism, and the circadian clock has been suggested to contribute to enhanced plant fitness by balancing innate immune responses with cellular metabolism [22, 23].

In *Arabidopsis*, the circadian clock consists of a series of intertwined feedback loops, regulated both transcriptionally and post-transcriptionally, through post-translational modification and protein turnover [8]. This mechanism appears to be conserved across plant species [24]. The circadian clock has been found to influence a variety of metabolic functions in the plant including chlorophyll biosynthesis, transport photosystems, starch synthesis and degradation, and nitrogen and sulfur assimilation [25]. The concentrations of metabolites including nitrate, glutamate, glutamine, and sucrose have also been shown to alter clock timing [26, 27]. However, due to differences in methodology, these results are sometimes inconsistent across studies, highlighting a need to consider photoperiod duration and the time of sample collection when describing results [25].

While the circadian clock machinery and associated metabolic and physiological responses have been well characterized, especially in *Arabidopsis* [28], little is known about how the rhizosphere bacterial community responds to host circadian cycling. Here, we examine the rhizosphere community dynamics of *Arabidopsis thaliana* in natural soil using next-generation sequencing (NGS) of the 16S rRNA gene. Soil organic matter (SOM) composition in the rhizosphere was also characterized by high-resolution mass spectrometry, 21T Fourier transform ion cyclotron resonance mass spectrometry (FTICR-MS), to help elucidate microbial metabolism.

In this study, we assessed cycling dynamics: (1) among biological replicates of *A. thaliana* sampled during dark and light periods to determine whether microbial community composition changed due to a diel cycle associated with exposure to light; (2) over a 72-h period utilizing an acyclic *Arabidopsis* line in which the *cca1* gene is ectopically over-expressed [29] to determine differences in rhizosphere communities between the wild-type and mutant genotypes; and (3) of rhizosphere communities of a second species, *Brachypodium distachyon*, to determine if this cycling phenomenon is conserved among physiologically diverse plant species. While current hypotheses suggest that microbial populations in the rhizosphere are relatively static [2, 3], our results suggest that the rhizosphere microbial community is surprisingly dynamic, responding to both biotic and abiotic factors, including the circadian clock.

## Results

### 16S rRNA characterization of AM and PM rhizosphere communities

Sequencing of the V5–V6 hypervariable regions of the 16S rRNA gene from DNA obtained from dark (AM, 1 h before light exposure) and light (PM, 1 h before dark) wild-type (Col-0) rhizosphere and fallow soil samples produced a mean Good’s coverage of 94.2 ± 2.3% (mean ± standard deviation), following rarefaction to 20,000 reads per sample with OTU binning at 97% similarity. While differences in alpha diversity, measured as Shannon diversity and abundance-based coverage estimate (ACE) of richness, did not significantly differ between dark and light samples (9 h between time points) for a given host environment (Table 1), Shannon diversity was significantly greater in rhizosphere samples versus fallow soil (*P* = 0.001). Samples had similar taxonomic compositions and were predominantly comprised of members of the *Proteobacteria*, *Actinobacteria*, and *Acidobacteria* (Additional file 1: Figure S1). Soil communities harbored greater relative abundances of the *Actinobacteria* and *Acidobacteria* than were observed in the rhizosphere. Communities in both the rhizosphere and fallow soil consisted of a large number (265 to 274) of families present at relatively low abundances (Additional file 1: Figure S2). Rhizosphere communities contained approximately 10 more families at low abundance than those of fallow soil. *Planctomycetaceae* was the most abundant family in both environments at both time points (Additional file 1: Figure S2), and about a quarter of the community (23.3 ± 2.3% and 28.5 ± 4.7% of sequence reads in rhizosphere and soil samples, respectively) could not be classified to a family.

**Table 1:** Coverage and alpha diversity (mean ± standard deviation) among AM and PM rhizosphere samples based on 16S rRNA gene sequencing

Host environment	Time	Number	Coverage (%)	*S*_obs_^a^	Shannon^b^	ACE
Wild-type	AM (dark)	10	94.5 ± 3.3	3120 ± 887	7.18 ± 0.22^A^	4771 ± 2620
	PM (light)	9	93.4 ± 1.9	3543 ± 587	7.29 ± 0.14^A^	4995 ± 1150
Fallow soil	AM (dark)	10	95.2 ± 1.9	2823 ± 536	6.89 ± 0.30^B^	4072 ± 1558
	PM (light)	10	93.5 ± 1.4	3290 ± 344	7.07 ± 0.11^A,B^	5272 ± 1335
				*P* value	0.001	0.486

Values sharing the same capital letter subscripts did not differ significantly by Tukey's *post-hoc* test (*P* < 0.05)

^a^ *S*_obs_: number of OTUs observed

^b^Indices sharing the same superscript did not differ significantly by Tukey’s post hoc test (*P* > 0.05)

Principal coordinate analysis of Bray-Curtis distances revealed significant separation (AMOVA, *F*_*s*_ = 1.28, *P* = 0.003) of dark (AM) communities from those characterized from light (PM) samples in the *Arabidopsis* rhizosphere (Additional file 1: Figure S3). Thus, while alpha diversity (species richness and evenness within a single sample) did not differ, community composition changed significantly between samples collected during dark and light conditions in the rhizosphere. In contrast, separation was not significant in fallow soil (*F*_*s*_ = 1.48, *P* = 0.076). Similarly, phylogenetic differences in community composition (phylobetadiversity, described further in the “Methods” section), assessed using unweighted UniFrac distances, showed significant differences (*P* = 0.031) between the dark and light communities in the rhizosphere, but not in soil alone (*P* = 0.217), suggesting changes in the soil were evolutionary driven.

**Fig. 1:**
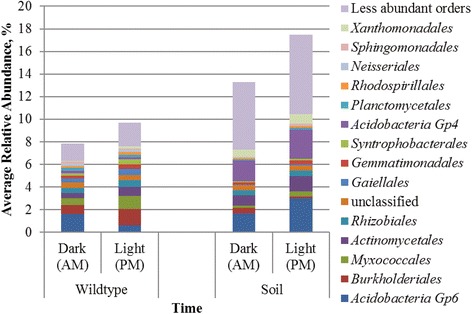
Order-level classification and relative abundances of OTUs that differed significantly between time points. More specific taxonomic classifications were not performed due to a large number of unclassified sequences found at family level. Significance was evaluated by using the Kruskal-Wallis test (*P* < 0.05). Analyses were performed separately for rhizosphere and fallow soil communities

Community compositions (beta diversity) between dark and light samples were significantly different in both environments (*P* = 0.018 and 0.031, for rhizosphere and soil communities, respectively), as evaluated by analysis of similarity (ANOSIM), but as stated above, phylobetadiversity did not differ in fallow soil. Among rhizosphere samples, <10% of the community showed variation in the relative abundances of operational taxonomic units (OTUs) between sampling times, with a significantly lower abundance of *Acidobacteria* Gp 6, and greater relative abundances of *Burkholderiales* and *Myxococcales*, among other orders (Fig. 1) between dark and light time points. The soil community showed greater variability between light and dark time points (13.3–17.5% of the community), with the most dynamic shifts observed within orders of the *Acidobacteria* (Fig. 1). Taken together, these results suggest that while beta diversity changed in both the rhizosphere and fallow soil between dark and light samples, only rhizosphere communities, and not those in soil, showed a significant change in phylobetadiversity, despite a greater percentage of community variation in soil.

### SOM characterization of AM and PM rhizosphere organic matter

**Fig. 2:**
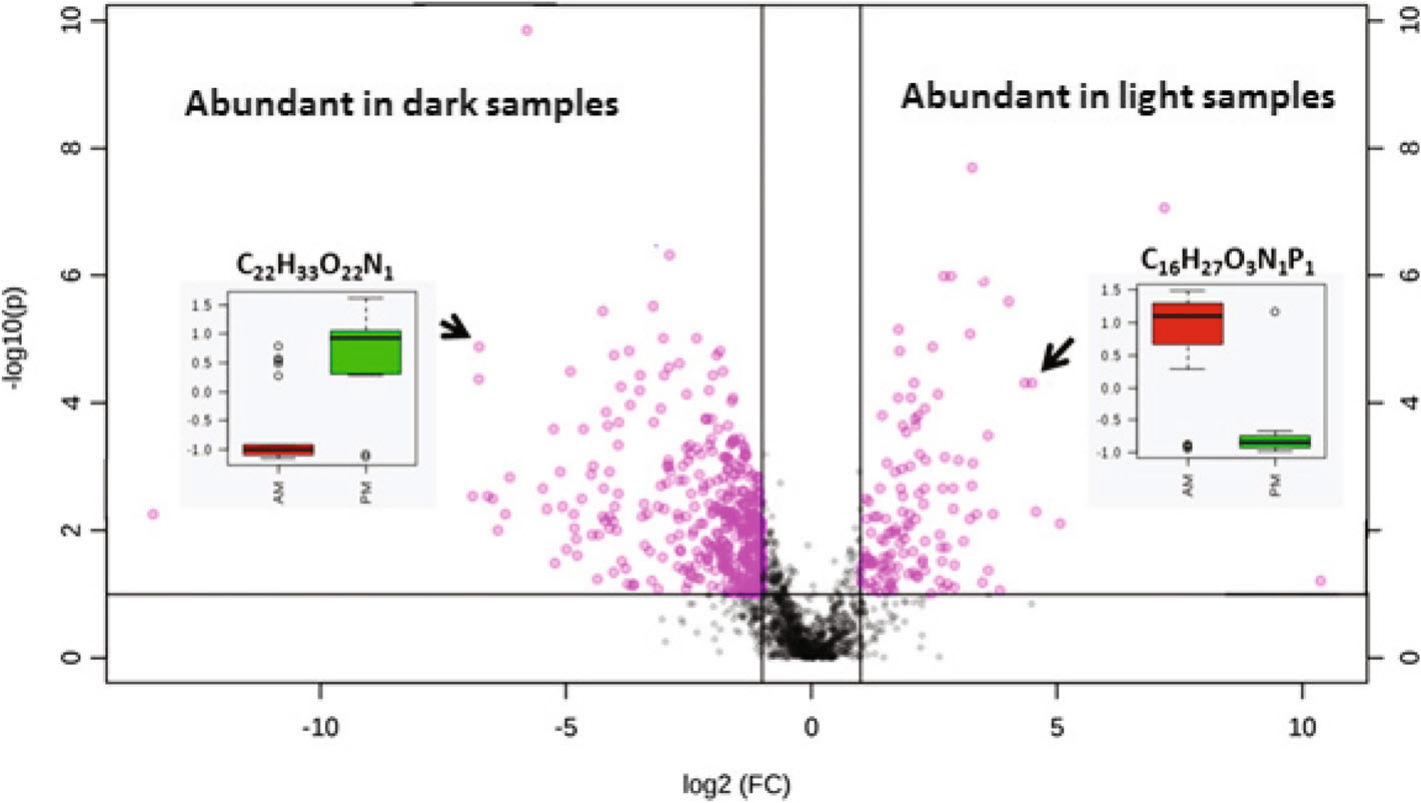
Relative abundances of different organic compounds identified by 21T FT-ICR-MS. The volcano plot was obtained by plotting the log_2_ fold change on the *x*-axis and –log_10_(*P*) on the *y*-axis. Compounds that changed twofold or more with a *P* value <0.05 are indicated in pink

The SOM composition was significantly different in the water fraction of rhizosphere samples between the dark (AM) and light (PM) samples (Fig. 2). Approximately two-fold formula-assigned compounds were more abundant among light samples compared to dark samples. Ordination of chemical compounds by principal components analysis (PCA, Additional file 1: Figure S4, Panel A and Figure S5, Panel A) revealed that dark and the light samples were mainly separated along PC1, whereas the different biological and technical replicates within each cluster were separated along PC2.

The organic compounds responsible for such separation were extracted from the loading plots and plotted on a van Krevelen diagram (Additional file 1: Figure S4, Panel B and Figure S5, Panel B). The majority of the compounds present in the light samples had high oxygen/carbon (O/C) ratios that fell in the aromatic-like, lignin-like, and tannin-like region of the van Krevelen diagram and represent potential alkaloids, ketones, terpenoids, flavonoids, organic acids, and condensed tannins. Additionally, the majority of these compounds included heteroatoms such as S, N, and P. On the contrary, compounds with low O/C and high hydrogen/carbon (H/C) ratios dominated the dark samples and mainly fit in the lipid-like and unsaturated H/C region of the van Krevelen diagrams. The majority of these compounds contained only C, H, and O. The increase in the abundance of lipid-like compounds in the dark samples compared to the light samples was significant and primarily drove the separation between the dark and light in methanol extracts (Additional file 1: Figure S5).

The average mass-to-charge ratio (*m/z*) range of the compounds identified in AM samples was lower than that in the PM samples, where the PM samples appeared to have significantly higher *m*/*z* values. Similarly, organic compounds in the PM samples had significantly higher nominal oxidation state of carbon (NOSC) values. Expressing the average oxidation state of all carbons in one formula, NOSC provides information on the biogeochemical reactivity of a compound. An increase in the NOSC values during the day is therefore consistent with increased oxidation and the production of soluble compounds that are available for microbial metabolism.

### Diurnal cycling of bacterial communities in the *Arabidopsis* rhizosphere

To further investigate potential cycling dynamics, bacterial communities were characterized every 6 h over a 72-h period from the rhizosphere of wild-type (Col-0) *Arabidopsis*, the rhizosphere of an acyclic line (OX34), and fallow soil. Samples were collected during dark conditions (1 am), 2 h before light (7 am), during light exposure (1 pm), and 1 h after dark (7 pm). Alpha diversity among samples from the wild-type rhizosphere, by both Shannon and ACE indices, was significantly greater (*P* = 0.018 and 0.024) during dark periods (1 am and 7 am samples), compared to when samples were exposed to light or shortly thereafter (1 pm and 7 pm samples) (Table 2). This apparent discrepancy from the earlier AM/PM experiment may reflect greater statistical power in this experiment due to a greater number of samples collected (9–10 compared to 17 in dark and light groups) or slight differences in the times when samples were collected relative to the photoperiod. Differences in alpha diversity between light and dark exposures were not significant among communities in the OX34 rhizosphere (*P* ≥ 0.813). In soil alone, differences in the Shannon index were also not significant (*P* = 0.091). In contrast, ACE richness was significantly greater among dark samples (*P* < 0.0001), which may reflect greater variation in soil over a longer time periods compared to single-time point AM/PM samples. Differences in ACE index at individual time points were also observed for wild-type rhizosphere and fallow soil (Table 2).

**Table 2:** Coverage and alpha diversity (mean ± standard deviation) among microbiota from the rhizosphere of wild-type and OX34 mutant *Arabidopsis* plants and fallow soil samples

Host environment	Time	*N* ^a^	Coverage (%)	*S*_obs_	Shannon^b^	ACE
Wild-type	1 am	6	96.4 ± 1.5	2656 ± 429	6.72 ± 0.15^A^	3420 ± 957^A,B^
	7 am	11	95.0 ± 1.0	3035 ± 230	6.83 ± 0.08^A^	4455 ± 1195^A^
	1 pm	8	97.5 ± 1.4	2278 ± 553	6.67 ± 0.21^A^	2714 ± 916^B^
	7 pm	9	97.2 ± 2.0	2372 ± 579	6.62 ± 0.22^A^	3185 ± 1990^A,B^
OX34 mutant	1 am	8	93.7 ± 1.1	3317 ± 308	6.87 ± 0.14^A^	5915 ± 1489^A^
	7 am	11	93.9 ± 1.2	3340 ± 327	6.89 ± 0.14^A^	5695 ± 1573^A^
	1 pm	9	93.7 ± 1.1	3357 ± 287	6.87 ± 0.11^A^	6020 ± 1348^A^
	7 pm	9	94.2 ± 1.8	3246 ± 388	6.87 ± 0.09^A^	5562 ± 2489^A^
Fallow soil	1 am	7	94.3 ± 1.8	3081 ± 483	6.82 ± 0.14^A^	5699 ± 2183^A,B^
	7 am	5	92.6 ± 0.9	3533 ± 218	6.93 ± 0.12^A^	7673 ± 1391^A^
	1 pm	6	96.3 ± 2.1	2540 ± 730	6.72 ± 0.20^A^	3449 ± 1354^B^
	7 pm	6	96.2 ± 1.3	2747 ± 328	6.82 ± 0.14^A^	3571 ± 769^B^

^a^ *N* refers to the total numbers of samples (replicates) collected at the time point over a 3-day period. Three samplings each were performed in triplicate at 1 am, 1 pm, and 7 pm, and four samplings were performed at 7 am. Samples that could not be rarefied to 22,380 sequences were removed from the dataset

^b^Times sharing the same superscript did not differ significantly by post hoc test for a given host environment (*P* > 0.05)

**Fig. 3:**
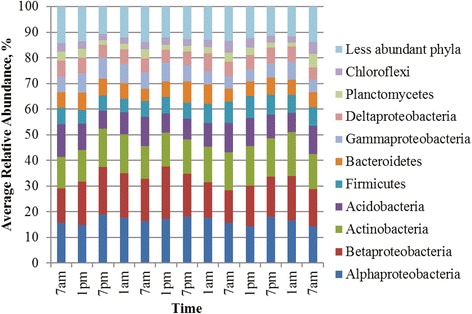
Distribution of phyla and *Proteobacteria *classes in rhizosphere samples from wild-type *Arabidopsis* plants. Percent relative abundance refers to cumulative abundance and 42 less abundant taxa that are not shown

Similar to the AM/PM experiment, bacterial communities among rhizosphere and soil samples were primarily comprised of members of the phyla *Proteobacteria*, *Bacteroidetes*, *Acidobacteria*, and *Actinobacteria* (Fig. 3 and Additional file 1: S6). Differences in beta diversity among host environments were significant by ANOSIM (*P* < 0.001), with most of the variation attributable to OTU-level shifts in relative abundance within the same predominant families in all environments (Additional file 1: Figure S7).

Increasing the duration of sampling revealed that bacterial communities collected from the wild-type rhizosphere (Fig. 3) appeared to show cycling in the relative abundances of phyla that corresponded to the 9-h photoperiod. Cycling patterns were generally characterized by increases in the *Proteobacteria*, except for the *Betaproteobacteria*, the *Actinobacteria*, and the *Firmicutes*, and decreases in the relative abundance of *Acidobacteria* and *Bacteroidetes* during light exposure. In contrast, less apparent diel variation was observed in the rhizosphere microbial communities obtained from the acyclic OX34 mutant (Additional file 1: Figure S6, Panel A).

**Fig. 4:**
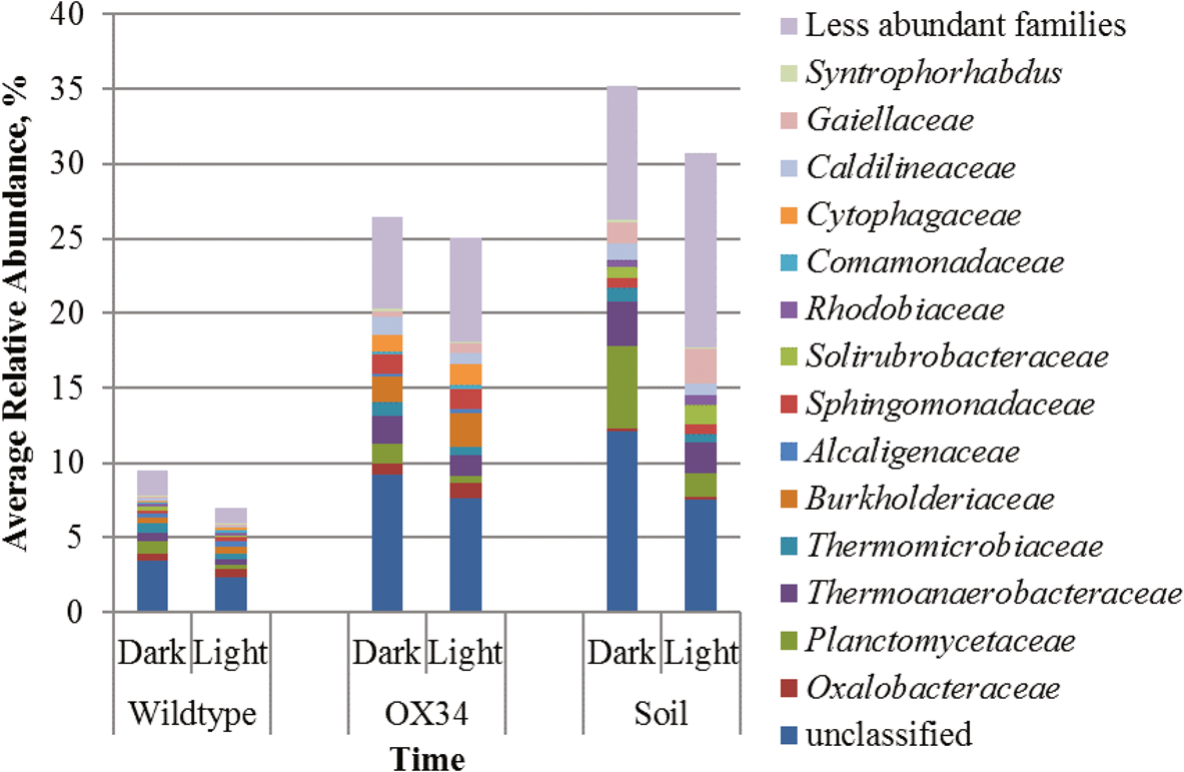
Family-level classification of OTUs that differed significantly among light and dark periods. Significance was evaluated by Kruskal-Wallis test (*P* < 0.05). Analyses were performed separately for each genotype in the *Arabidopsis* rhizosphere and fallow soil

Cycling dynamics among the rhizosphere genotypes and fallow soil were further statistically interrogated using the Jonckheere-Terpstra-Kendall (JTK) algorithm [30], with OTUs assigned to families. By this analysis, a significantly greater proportion of the community (*P* < 0.0001) exhibited cycling dynamics in the wild-type rhizosphere (13.2 ± 1.1%), compared to that obtained from the OX34 mutant rhizosphere (3.6 ± 0.7%) or fallow soil (1.3 ± 0.7%). *Burkholderiaceae*, *Rhodospirillaceae*, *Planctomycetaceae*, and *Gaiellaceae* were among the most abundant cycling families identified. Similarly, the Kruskal-Wallis test indicated that OTUs that varied between light and dark periods, without determination of cycling dynamics, accounted for up to 9.5%, on average, of the community in the wild-type rhizosphere (Fig. 4), and these OTUs were classified to several families found to exhibit cycling dynamics by the JTK algorithm. The microbial community in the OX34 rhizosphere and fallow soil showed greater amounts of variability independent of cycling by Kruskal-Wallis test, with ~25% of the community varying in the mutant rhizosphere and 30–35% varying in soil alone. Thus, the key feature between the wild type, OX34, and soil was that, although some variability was seen in each sample type, the wild type showed a greater statistically significant cycling effect, encompassing a greater proportion of the community.

As described in detail in Additional file 1, a similar experiment was performed in which significant (*P* < 0.05) diurnal cycling of the rhizosphere bacterial community associated with *B. distachyon* was also observed. In contrast to *Arabidopsis*, there was no significant difference in alpha diversity between light and dark periods (*P* = 0.658). However, 3.5% of the community showed significant (*P* < 0.05) cycling dynamics using the JTK algorithm, the most abundant of which was *Gaiellaceae*. In contrast, only 0.2% of the community, on average, in fallow soil showed significant cycling dynamics. Similar to *Arabidopsis*, a similar percentage of the community (<12%) also varied between light and dark periods by Kruskal-Wallis test.

### Cycling of inferred functional genes

**Fig. 5:**
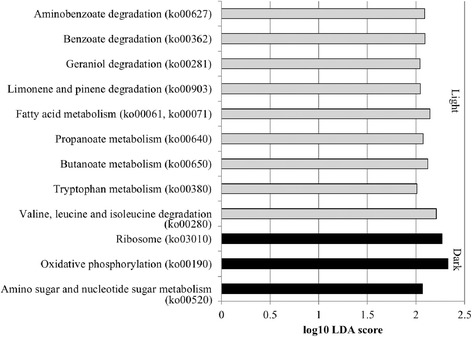
Tier 3 KO inferred functional annotations that differed by LEfSe analysis. Inferred functions differed between bacterial communities from wild-type *Arabidopsis* rhizosphere samples harvested in AM and PM time points

Changes in potential metabolic functions as a result of taxonomic cycling were assessed using PICRUSt [31] to infer abundances of functional genes. Twelve inferred tier 3 KEGG orthology (KO) categories differed between time points among dark (AM) and light (PM) wild-type *Arabidopsis* rhizosphere samples, predominantly within the broader category of metabolism (Fig. 5). Generally, only one or two taxa were found to contribute at high abundances to these functional categories, particularly members of the families *Nitrospiraceae* and *Comamonadaceae* (Additional file 1: Table S1). In contrast, no functional categories differed between dark and light time points in fallow soil.

The JTK algorithm showed that 23 tier 3 KO functional categories had significant cycling among wild-type *Arabidopsis* rhizosphere bacterial communities throughout the 72-h experiment (inferred abundances supplied in Additional file 1: Table S2). Functional predictions were assigned within the broader category of metabolism (Additional file 1: Figure S8), with genes encoding functions associated with carbohydrate metabolism showing the greatest abundances 1 h before exposure to light (7 am). Due to the high diversity of taxa associated with carbohydrate metabolism among Col-O samples, taxonomic affiliations of OTUs associated with these fluctuations could not be definitively determined. In contrast, the lowest abundances were observed in the middle of the dark period (1 am). Cycling of “ether lipid metabolism” (ko00565), “glycosphingolipid biosynthesis - lacto and neolacto series” (ko00564), and “neuroactive ligand-receptor interaction” (ko04080) were also identified using the JTK algorithm in the OX34 rhizosphere, albeit at low abundance. No significant cycling of functional genes in fallow soil could be identified using the JTK algorithm.

## Discussion

Results of this study showed that rhizosphere microbiota composition and predicted function varied according to a diel cycle and differed between the acyclic OX34 line and wild-type plants. Approximately 10% of the bacterial community in the wild-type *Arabidopsis* rhizosphere, determined by the JTK, exhibited diel cycling that was not seen in the acyclic *Arabidopsis* line. This strongly suggests that there is a relationship between changes in plant metabolism and bacterial community composition. Similar to previous studies [2, 3], the same predominant phyla were observed in rhizosphere and soil samples, and the relatively high percentage of taxonomically unclassified OTUs is also similar to previous findings [2]. Notably, many families previously found to be associated with root communities, for which the plant would likely select [2] (e.g., *Flavobacteriaceae*, *Rhizobiaceae*, *Comamonadaceae*, and *Oxalobacteraceae*), were differentially abundant between light and dark periods or showed significant cycling dynamics. This suggests that these potentially selected taxa are responding to host circadian-induced changes or may be contributing to them.

Species showing cyclical variation in abundances may also serve as pioneer microorganisms colonizing newly emerging root tissue. Root growth and elongation is controlled by the circadian clock in *Arabidopsis*, with faster growth reported under light and during longer light conditions [32]. Similarly, previously reported root-associated taxa, including members of the *Actinomycetales*, *Burkholderiales*, and *Flavobacteriales* [2, 33], among others, were observed to increase in abundance during light periods. Furthermore, variation in beta diversity was reduced during light conditions (demonstrated by closer clustering, shown in Additional file 1: Figure S3), which may indicate a selective pressure exerted by the root community to promote a community composition that facilitates plant growth. While this suggestion cannot be definitively concluded from the data presented here, follow-up studies using fluorescent in situ hybridization may help elucidate the dynamics of the root microbiota as they relate to regions of greater root growth (root tips).

Plant carbon partitioning may directly influence microbial associations, and this partitioning also shows diel fluctuations and is regulated by the circadian clock [34]. This likely defines a “metabolic dawn,” where sugar from photosynthesis may feed back to the clock to set its rhythm on a daily basis [35]. Soluble sugars [36], as well as genes associated with starch synthesis, chloroplast biosynthesis, and photosystems [25, 34], peak at or approximately 4 h after dawn, presumably to allow maximal production of sugars during the light period, a fraction of which are released to soil [37]. These results are consistent with SOM composition derived from high-resolution mass spectra that indicated an increase in labile compounds such as sugars during the light period. In contrast, genes regulating transport, breakdown, and storage of sugars, glycolysis, and the pentose phosphate pathway reach their maximum toward dusk to maximize the mobilization of starch and maintain carbon homeostasis [34]. Interestingly, among the rhizosphere bacterial community, genes involved in glycolysis and the pentose phosphate pathway were similarly inferred to show cycling dynamics (Additional file 1: Figure S8) in opposite phase to that previously observed in *Arabidopsis* [25, 34]. Thus, one attractive hypothesis is that the plant and rhizosphere communities are communicating to serve complementary functions, mediated in large part by circadian cycling.

A very small percentage of the rhizosphere bacterial community showed cycling dynamics in fallow soil samples. Both photosynthetic and non-photosynthetic bacteria have previously been shown to exhibit their own circadian rhythms, entrained by environmental conditions [38–40]. Thus, this fluctuation may represent independent cycling of these bacteria or changes in water potential due to plant transpiration.

The magnitude of community variation determined by Kruskal-Wallis test, unrelated to cycling dynamics, varied considerably between wild-type and mutant rhizosphere samples and fallow soil. Mutant rhizosphere and soil communities showed greater variation, independent of cycling, than wild-type rhizosphere communities, yet a smaller proportion of the community showed significant cycling dynamics by the JTK algorithm, suggesting that variation in mutant rhizosphere and soil samples is likely stochastic, rather than cyclical. Furthermore, while the composition of dark and light communities in fallow soil was significantly different, phylogenetic structure of the community was not significantly altered, although it was in wild-type rhizosphere samples. This result suggests that shifts in the wild-type rhizosphere are more likely to be directed rather than as a result of stochastic variation, resulting in a phylogenetic restructuring of the community. Lower variation between light and dark sampling points in both wild-type and mutant rhizosphere samples may also likely reflect moderation of the community by rhizosphere effects [4]. Fewer cycling taxa were observed in the rhizosphere community of *Brachypodium*, relative to *Arabidopsis*, although a significantly greater percentage of the rhizosphere community showed cycling dynamics relative to fallow soil. Differences between *Arabidopsis* and *Brachypodium* cycling communities are not surprising since species-specific microbial assemblages are determined based on the nature of root exudates and plant-microbe signaling molecules [2, 4]. Thus, difference in assemblage may, in large part, help explain differences in cycling dynamics observed between species.

It is also important to note that functional genes reported here were inferred from taxonomic data [31]. Nevertheless, the taxa associated with diurnal cycling in this study are among those previously found to be selected for in *Arabidopsis* root communities [2, 3, 33] and the functional genes inferred corresponded reasonably well to previously reported gene expression data for *Arabidopsis* [34]. This further supports the contention that there is a true rhizosphere cycling dynamic that is important to the health and growth of the plant.

It is likely that there is two-way communication between the plant and its microbiota and that the rhizosphere microbial community influences circadian rhythm, gene expression, and metabolic functioning of the host plant. Microbial regulation of host genes has been observed in rhizobial-legume interactions and in the *Euprymna scolopes* squid, in which *Vibrio fischeri* bioluminescence regulates expression of a host cryptochrome [41]. Similarly, the gut microbiota regulates rhythmic signaling pathways in intestinal epithelial cells that coordinate glucocorticoid production in the intestine [42], and the bacterial community is responsible for normal clock functioning in the liver and hypothalamus in mice [9]. Several groups have begun investigating how rhizosphere and endophytic communities might be used to improve plant biomass and stress resistance [6], and future studies in *Arabidopsis* are necessary to understand how the microbiota influences host plant circadian functioning and productivity.

## Conclusions

The extent of change of the rhizosphere microbiota over a 24-h period equals or exceeds those previously attributed to factors such as plant genotype [43–45]. While current hypotheses suggest that microbial populations in the rhizosphere are relatively static and primarily related to soil communities [2, 3, 33], our work suggests otherwise. The rhizosphere microbial community indeed appears to be very dynamic in time, responding to both biotic and abiotic factors. This additional complexity may shape a better understanding of plant-microbe interactions and how rhizosphere dynamics may affect plant productivity in a changing environment. Perhaps, more importantly, our results highlight that temporal factors including light exposure, in addition to other sources of experimental error, should be considered in the interpretation of data from future studies, since in several previous studies, the time of sampling is either not noted or not well controlled. Expanding upon the scope of the present work, which was conducted using an annual dicot, to include perennial plants and most particularly bioenergy grasses, will allow us to consider new possibilities of plant-microbial interactions.

## Methods

### Rhizosphere sample harvesting

Rhizosphere soil samples were obtained as previously described [46] after plants had grown in soil for 5 weeks. Three experiments were conducted: (1) a biological replicate experiment in which *A. thaliana* or fallow soil (*n* = 10 for each) was collected 1 h prior to light exposure (AM) and 1 h prior to turning off the lights (PM), with a 9-h photoperiod (9 am–6 pm); (2) a 72-h cycling experiment in which wild-type *Arabidopsis*, the *acc1*-ox34 (OX34) acyclic mutant strain of *Arabidopsis* [47], or fallow soil was grown with a 9-h light period (9 am–6 pm) and harvested in triplicate every 6 h (7 am, 1 pm, 7 pm, and 1 am) for 72 h; and (3) a 72-h *B. distachyon* BD21 experiment that employed the same sampling regime as experiment 2, except using *Brachypodium* and fallow soil with a 12-h light period (8 am–8 pm). A 9-h photoperiod was used for *Arabidopsis* experiments to avoid flowering prior to sample collection, while a 12-h photoperiod was used for the *Brachypodium* experiment to allow optimal growth.

For the AM/PM experiment, 10 replicates were harvested 1 h prior to light exposure (AM) and 1 h prior to turning off the lights (PM). Each replicate contained rhizosphere soil from 25 plants (fallow soil replicates were collected from 10 individual pots containing only soil located in the same growth chamber with plants). For the 72-h cycling experiments of *Arabidopsis* or *Brachypodium*, three replicates were collected for each time point and each replicate was collected from ≥30 plants. Loose soil was manually removed from the roots by shaking with sterile gloves. Roots were placed in a clean and sterile 50-ml tube containing 22.5 ml of ice-cold extraction buffer (0.1% sodium pyrophosphate, pH 7.0, and 0.1% Tween 20) and 2.5 ml of RNA stop solution (5% water-saturated phenol, pH 4.3, in 95% ethanol). Tubes were vortexed at maximum speed for 20 s, which released most of the rhizosphere soil from the roots and turned the water turbid. The turbid solution was transferred onto a petri dish, and sterile forceps were used to remove broken plant parts followed by filtering the mixture through a Miracloth strainer into a new 50-ml tube. The turbid filtrate was centrifuged for 15 min at 3200×*g* to form a pellet containing fine sediment and microorganisms. Most of the supernatant was removed and the loose pellets were resuspended and transferred to 2-ml microfuge tubes. The tubes were centrifuged at 10,000×*g* for 5 min to form tight pellets, from which all supernatant was removed. The final rhizosphere pellets were flash-frozen in liquid nitrogen and stored at −80 °C until used.

### SOM extraction and characterization

Two different solvents with different polarities in the order of (1) water-H2O and (2) methanol-CH3OH were used to sequentially extract organic matter (OM) from the rhizosphere soil, as described previously [48]. Briefly, samples were prepared by adding 0.6 ml of solvent to 50 mg soil and shaking for 2 h at room temperature followed by mixing at 800 rpm on an Eppendorf Thermomixer (Eppendorf, Hauppauge, NY, USA) in 2 ml capped glass vials. Samples were removed from the shaker and centrifuged for 16 min at 3500 rpm, and the supernatant was removed. The soil residue was dried with nitrogen gas and the second solvent was added and processed in the same way. The extracts were then injected directly into the 21T Agilent FTICR mass spectrometer (magnet from Agilent Technologies, Oxford, England; spectrometer built at the Environmental Molecular Sciences Laboratory (EMSL)) using a Hamilton 250 μl glass syringe (Hamilton Company, Reno, Nevada, USA) at a flow rate of 0.5 μl min^−1^. The H_2_O extracts were diluted in MeOH at a ratio of 1:2 to improve electrospray ionization (ESI) efficiency.

Mass spectrometry was performed by the EMSL. Samples were introduced directly to a 21T FTICR mass spectrometer (Thermo Scientific, San Jose, CA, USA) outfitted with a custom ESI interface. Electrospray emitters were custom made using 360 μm outer diameter × 50 μm inner diameter, chemically etched, fused silica, as described previously [49]. The ion transfer tube temperature and spray voltage were 300 °C and 3.0 kV, respectively. Mass spectra (AGC 3 × 10^6^) were collected from 240 to 1200 *m*/*z* with a mass measurement accuracy of less than 500 ppb. Four hundred individual scans were averaged for each sample and internally calibrated using organic matter homologous series separated by 14 Da (–CH_2_ groups). Chemical formulas were assigned using in-house software based on the compound identification algorithm (CIA), described by Kujawinski and Behn [50] and modified by Minor et al. [51]. Chemical formulas were assigned based on the following criteria: S/N >2, and mass measurement error <0.5 ppm, taking into consideration the presence of C, H, O, N, S, and P and excluding other elements.

To interpret the large data set, the chemical character of thousands of data points for each sample spectrum were evaluated on van Krevelen diagrams generated from the complex mass spectra obtained by ESI FTICR MS [52]. Van Krevelen diagrams provide a means to visualize and compare the average properties of OM and enable identification of the major biochemical classes (i.e., lipids, proteins, lignin, carbohydrates, and condensed aromatics) of compounds present in samples. Compounds were assigned on the van Krevelen diagram on the basis of their molar H/C ratios (*y*-axis) and molar O/C ratios (*x*-axis). The stoichiometry of each assigned formula was used to calculate nominal oxidation state of carbon (NOSC) for each compound [53]. NOSC of individual compounds present in each sample was averaged to give the NOSC of soluble organic matter in that sample. In addition, MetaboAnalyst 3.0 [54] was used to generate volcano and principal component analysis (PCA) plots. We only considered compounds with assigned molecular formulae because we wanted to extract information with respect to SOM chemical characteristics. Additionally, only peaks that were present in more than 50% of the replicates were included in such analysis, and the ICR-MS data were both normalized (by median) and scaled (pareto scaling) to emphasize the importance of the compounds with smaller relative intensities using MetaboAnalyst.

### DNA extraction and sequencing

DNA was extracted from 200 to 300 mg rhizosphere or soil samples using the PowerSoil^®^ DNA Isolation Kit (MoBio Laboratories, Inc, Carlsbad, CA, USA) according to the manufacturer’s instructions. The V5 + V6 regions of the 16S rRNA gene were amplified using the barcoded BSF784/1064R primer set [55, 56] with a negative (sterile water) control by the University of Minnesota Genomics Center (UMGC, Minneapolis, MN, USA). Amplicons were gel purified, pooled in equal amounts, and paired-end sequenced at a read length of 300 nt on the Illumina MiSeq platform (Illumina, Inc., San Diego, CA, USA) by UMGC.

### Bioinformatics

All sequence processing was done using mothur ver. 1.34.0 [57]. Sequences were trimmed to the first 150 nt to remove low-quality regions at the ends of reads, and reads were paired-end joined using fastq-join software [58]. Joined reads were trimmed to maintain an average quality score of at least 35 over a sliding 50 nt window, and sequences with more than two mismatches from primer sequences, >8 nt homopolymers, and those with ambiguous bases were removed. High-quality reads were aligned against the SILVA database ver. 119 [59] and subjected to a 2% pre-cluster step to remove probable sequence errors [60]. Chimeric sequences were identified and removed using UCHIME software [61]. Samples were rarefied to 20,000, 22,380, and 33,685 sequence reads, with respect to experiments 1, 2, and 3, respectively, by random subsample for further comparison and analysis [62].

Operational taxonomic units were assigned at 97% identity using the furthest-neighbor algorithm and taxonomic assignments were performed against ver. 14 of the Ribosomal Database Project [63]. For functional predictions, taxonomic classification was performed against the GreenGenes database ver. 13.5 [64]. Functional inferences within the Kyoto Encyclopedia of Genes and Genomes (KEGG) orthology were made using PICRUSt (*p*hylogenetic *i*nvestigation of *c*ommunities by *r*econstruction of unobserved *st*ates) software [31], with normalization to 16S rRNA gene copy number.

### Statistical analyses

Statistical analyses of diversity, community composition (beta diversity), phylogenetic structures (phylobetadiversity), and ordination were performed using mothur. Alpha diversity was calculated using the Shannon and abundance-based coverage (ACE) indices. Differences in beta diversity were evaluated using analysis of similarity (ANOSIM) [65], phylobetadiversity was analyzed using unweighted Unifrac distances [66], and cluster analysis was performed using analysis of molecular variance (AMOVA) [67] using Bray-Curtis dissimilarity matrices [68]. In this study, we used the terminology of Graham and Fine [69] to define beta diversity as a change in the species composition between samples while phylobetadiversity referred to a change in phylogenetic relatedness of communities.

Ordination of samples was performed via principal coordinate analysis. To determine which members of the community exhibited significant cycling in relative abundance during light and dark cycles, community composition was evaluated at the family level using the JTK algorithm [30] in R ver. 3.2.2 [70]. Zeit geber time 0 was defined as the first sample collected at 7 am. Differences in functional inferences were evaluated using LEfSe [71], which employs consecutive Kruskal-Wallis and Wilcoxon rank-abundance tests and then utilizes linear discriminant analysis (LDA) to estimate effect sizes of features. Differences in OTUs were evaluated by Kruskal-Wallis test [72]. Other statistical analyses were performed using XLSTAT ver. 2015.01.0 (Addinsoft, Belmont, MA). All statistics were performed at *α* = 0.05.

## Additional file

Additional file 1: Table S1. Taxonomic classification of OTUs predominantly associated with tier 3 KO inferred functional annotations that differed between bacterial communities from wild-type *Arabidopsis* rhizosphere samples harvested in dark (AM) and light (PM) time points (Fig. 5). Functions for which contributing taxa could not be determined are not shown. **Table S2.** Functional annotations of inferred genes that show significant cycling in abundance in wild-type *Arabidopsis* rhizosphere communities. Functional categories are arranged by KEGG orthology tiers, with each tier increasing in specificity. **Table S3.** Alpha diversity indices for *Brachypodium distachyon* BD21 experiment samples. Figure S1 Distribution of abundant phyla among AM and PM samples in the rhizosphere of wild-type *Arabidopsis* and fallow soil. **Figure S2.** Distribution of abundant families among AM and PM samples in the rhizosphere of wild-type *Arabidopsis* and fallow soil. **Figure S3.** Principal coordinate analysis of Bray-Curtis dissimilarities among AM and PM bacterial communities characterized by 16S rRNA sequencing in A) the rhizosphere of *Arabidopsis* (*r* ^2^ = 0.689) and B) fallow soil (*r* ^2^ = 0.876). Points represent individual samples. **Figure S4.** PCA plot of the relative abundance of the different organic compounds identified in each sample by water extraction (A) and van Krevelen diagram of elemental H/C (hydrogen-to-carbon) vs O/C (oxygen-to-carbon) ratios of the organic compounds extracted from the loading plot of the PCA plot (B). **Figure S5.** 3D PCA plot of the relative abundance of the different compounds extracted by MeOH (A) and van Krevelen diagram of elemental H/C (hydrogen-to-carbon) vs O/C (oxygen-to-carbon) ratios of the organic compounds extracted from the loading plot of the PCA plot (B)). **Figure S6.** Distributions of phyla in (A) *Arabidopsis* OX34 mutant rhizosphere and (B) fallow soil samples, omitting less abundant phyla. Percent abundances are cumulative. Sequence data from later time points for soil could not be obtained, and less abundant taxa are not shown. **Figure S7.** Family-level classification of OTUs that differed significantly among *Arabidopsis* rhizosphere genotypes and soil groups by Kruskal-Wallis test (*P* < 0.05). **Figure S8.** Inferred carbohydrate metabolism genes that showed significant cycling (*P* < 0.05) in abundance among wild-type *Arabidopsis* rhizosphere bacterial communities by the JTK algorithm. **Figure S9.** Distribution of phyla among samples from (A) the *Brachypodium* rhizosphere and (B) fallow soil. Forty-one less abundant phyla are not shown. **Figure S10.** Family-level classification of OTUs that differed significantly among light and dark periods by Kruskal-Wallis test (*P* < 0.05) in the *Brachypodium* rhizosphere. (DOCX 980 kb).

## Abbreviations

ACE: Abundance-based coverage estimate
ANOSIM: Analysis of similarity
FTICR-MS: Fourier transform ion cyclotron resonance mass spectrometry
JTK: Jonckheere-Terpstra-Kendall algorithm
KO: Kyoto Encyclopedia of Genes and Genomes orthology
NGS: Next-generation sequencing
OTU: Operational taxonomic unit
PCA: Principal component analysis
SOM: Soil organic matter
