# Supplementary material for: Diurnal cycling of rhizosphere bacterial communities is associated with shifts in carbon metabolism
Source: Microbiome. 2017 Jun 24;5:65. doi: 10.1186/s40168-017-0287-1 (PMC5483260; doi:10.1186/s40168-017-0287-1)
Supplement: Additional file 1: Table S1. — Taxonomic classification of OTUs predominantly associated with tier 3 KO inferred functional annotations that differed between bacterial communities from wild-type Arabidopsis rhizosphere samples harvested in dark (AM) and light (PM) time points (Fig. 5). Functions for which contributing taxa could not be determined are not shown. Table S2. Functional annotations of inferred genes that show significant cycling in abundance in wild-type Arabidopsis rhizosphere communities. Functional categories are arranged by KEGG orthology tiers, with each tier increasing in specificity. Table S3. Alpha diversity indices for Brachypodium distachyon BD21 experiment samples. Figure S1 Distribution of abundant phyla among AM and PM samples in the rhizosphere of wild-type Arabidopsis and fallow soil. Figure S2. Distribution of abundant families among AM and PM samples in the rhizosphere of wild-type Arabidopsis and fallow soil. Figure S3. Principal coordinate analysis of Bray-Curtis dissimilarities among AM and PM bacterial communities characterized by 16S rRNA sequencing in A) the rhizosphere of Arabidopsis (r 2 = 0.689) and B) fallow soil (r 2 = 0.876). Points represent individual samples. Figure S4. PCA plot of the relative abundance of the different organic compounds identified in each sample by water extraction (A) and van Krevelen diagram of elemental H/C (hydrogen-to-carbon) vs O/C (oxygen-to-carbon) ratios of the organic compounds extracted from the loading plot of the PCA plot (B). Figure S5. 3D PCA plot of the relative abundance of the different compounds extracted by MeOH (A) and van Krevelen diagram of elemental H/C (hydrogen-to-carbon) vs O/C (oxygen-to-carbon) ratios of the organic compounds extracted from the loading plot of the PCA plot (B)). Figure S6. Distributions of phyla in (A) Arabidopsis OX34 mutant rhizosphere and (B) fallow soil samples, omitting less abundant phyla. Percent abundances are cumulative. Sequence data from later time points for soil could not be o [file 40168_2017_287_MOESM1_ESM.docx]

# Supplementary Results, Tables and Figures

# Circadian Cycling of Rhizosphere Bacterial Communities is Associated with Shifts in Carbon Metabolism

Christopher Staley^1^, Abigail P. Ferrieri^2^, Malak M. Tfaily^2^, Yaya Cui^3^, Rosalie K. Chu^2^, Ping Wang^1^, Jared B. Shaw^2^, Charles K. Ansong^4^, Heather Brewer^2^, Angela D. Norbeck^2^, Meng Markillie^2^, Fernanda do Amaral^3^, Thalita Tuleski^3^, Tomás Pellizzaro^3^, Beverly Agtuca^3^, Richard Ferrieri^5^, Susannah G. Tringe^6^, Ljiljana Paša-Tolić^2^, Gary Stacey^3,#^, and Michael J. Sadowsky^1,#^

^1^Biotechnology Institute, University of Minnesota, St. Paul, MN. 55108; ^2^Environmental and Molecular Sciences Laboratory, Pacific Northwest National Laboratory, Richland, WA, 99354; ^3^Division of Plant Science and Biochemistry, C.S. Bond Life Science Center, University of Missouri, Columbia, MO, 65211.^4^Biological Sciences Division, Pacific Northwest National Laboratory, Richland, WA, 99354; ^5^Department of Chemistry, University of Missouri Research Reactor, Columbia, MO, 65211; ^6^Microbial Systems Group, Metagenome Program, DOE Joint Genome Institute, Walnut Creek, CA, 94598;

^#^Corresponding Authors:

Ljiljana Paša-Tolić, Environmental Molecular Sciences Laboratory, Pacific Northwest National Laboratory, Richland, WA, 99354, Phone: (509)-371-6003; email: Ljiljana.PasaTolic@pnnl.gov

Michael J. Sadowsky, BioTechnology Institute, University of Minnesota, 140 Gortner Lab, 1479 Gortner Ave, Saint Paul, MN 55108; Phone: (612)-624-2706, Email: sadowsky@umn.edu

Running title: Circadian cycling of rhizosphere bacteria

Keywords: *Arabidopsis*, bacterial community structure, circadian rhythm, rhizosphere

## Supplementary Results

An experiment similar to the 72 h observation of *Arabidopsis* was carried out using *Brachypodium*, with a fallow soil control to evaluate potential bacterial cycling dynamics associated with other species. Triplicate samples, reflecting individual plants, were collected every 6 h, but the photoperiod was extended to 12 h to allow proper growth of *Brachypodium*. Each day, samples from two time points were collected during periods of light (1pm and 7pm) and dark (1am and 7am). Alpha diversity (Table S3), as measured by the Shannon index, was not significantly different among the time points for either rhizosphere (*P* = 0.658) or soil (*P* = 0.590). Similarly, ACE richness did not differ significantly by time in fallow soil (*P* = 0.427); however, among rhizosphere samples, ACE richness was significantly higher among samples collected during light exposure (*P* = 0.011).

The predominant phyla in the *Brachypodium* rhizosphere and soil communities were similar to those observed for *Arabidopsis* and were primarily comprised of Proteobacteria, Actinobacteria, Acidobacteria and Bacteroidetes (Figure S9). Similar to wildtype *Arabidopsis*, some cycling was observed in relative abundances of phyla in the rhizosphere that corresponded with the photoperiod (Figure S9, Panel A), but this was not the case in soil samples (Figure S9, Panel B). JTK analysis revealed that families, most prominently *Gaiellaceae*, significantly associated with cycling accounted for a lower percent of the community (3.5 ± 0.7%) than for *Arabidopsis*, and only a very small proportion of the soil community (0.2 ± 0.1%) was associated with cycling dynamics. Similarly, Kruskal-Wallis analysis revealed that a relatively small percent of the community (< 12%, Figure S10) differed in abundance among dark and light periods, and several of the families identified were also those found to exhibit significant cycling dynamics. In contrast, < 4.2% of fallow soil communities, on average, showed significant differences in OTU abundances between dark and light periods (data not shown).

Similar to taxonomic analyses, fewer inferred functional genes were found to show significant cycling in abundance in the *Brachypodium* rhizosphere. Only the genes involved in ‘clavulanic acid biosynthesis’ (ko00331) and ‘biosynthesis of type II polyketide backbone’ (ko01056) were found to cycle and abundances inferred were low (data not shown). In the soil, only one gene category associated with ‘signal transduction’ was found to cycle and also had very low abundance.

**Table S1 –** Taxonomic classification of OTUs predominantly associated with tier 3 KO inferred functional annotations that differed between bacterial communities from wild-type *Arabidopsis* rhizosphere samples harvested in dark (AM) and light (PM) time points (Figure 5). Functions for which contributing taxa could not be determined are not shown.

| Function | Predominant Taxon/Taxa |
| --- | --- |
| Aminobenzoate degradation (K00627) | Nitrospirae; Nitrospira; Nitrospirales; Nitrsospiraceae; Nitrospira  Proteobacteria; Betaproteobacteria; Burkholderiales; Comamonadaceae; Methylibium |
| Benzoate degradation (K00362) | Planctomycetes; Planctomycetia; Pirellulales; Pirellulaceae  Proteobacteria; Betaproteobacteria; Burkholderiales; Comamonadaceae; Methylibium |
| Geraniol degradation (K00281) | Proteobacteria; Betaproteobacteria; Burkholderiales; Comamonadaceae; Methylibium |
| Limonene and pinene degradation (K00903) | Acidobacteria; Acidobacteria-6  Gemmatimonadetes; Gemm-1 |
| Fatty acid metabolism (K00061/71) | Actinobacteria; Actinobacteria; Actinomycetales; Pseudonocardiaceae |
| Propanoate metabolism (K00640) | Nitrospirae; Nitrospira; Nitrospirales; Nitrsospiraceae; Nitrospira  Proteobacteria; Betaproteobacteria; Burkholderiales; Comamonadaceae; Methylibium |
| Tryptophan metabolism (K00380) | Planctomycetes; Planctomycetia; Pirellulales; Pirellulaceae  Proteobacteria; Betaproteobacteria; Burkholderiales; Comamonadaceae; Methylibium |
| Valine, leucine, and isoleucine degradation (K00280) | Actinobacteria; Actinobacteria; Actinomycetales; Propionibacteriaceae; Propionibacterium |
| Oxidation phosphorylation (K00190) | Acidobacteria; Acidobacteria-6 |
| Amino sugar and nucleotide sugar metabolism (K00520) | Nitrospirae; Nitrospira; Nitrospirales; Nitrsospiraceae; Nitrospira |

**Table S2** – Functional annotations of inferred genes that show significant cycling in abundance in wildtype *Arabidopsis* rhizosphere communities. Functional categories are arranged by KEGG orthology tiers, with each tier increasing in specificity.

| **KO Tier 1** | **KO Tier 2** | **KO Tier 3** |
| --- | --- | --- |
| Cellular Processes | Transport and Catabolism | Lysosome |
| Genetic Information Processing | Folding, Sorting and Degradation | Protein export |
| Environmental Information Processing | Signal Transduction | Notch signaling pathway |
| Environmental Information Processing | Signal Transduction | Wnt signaling pathway |
| Environmental Information Processing | Signaling Molecules and Interaction | Bacterial toxins |
| Metabolism | Amino Acid Metabolism | Tyrosine metabolism |
| Metabolism | Amino Acid Metabolism | Valine, leucine and isoleucine biosynthesis |
| Metabolism | Metabolism of Other Amino Acids | D-Arginine and D-ornithine metabolism |
| Metabolism | Metabolism of Other Amino Acids | Taurine and hypotaurine metabolism |
| Metabolism | Glycan Biosynthesis and Metabolism | Glycosaminoglycan degradation |
| Metabolism | Lipid Metabolism | Steroid hormone biosynthesis |
| Metabolism | Carbohydrate Metabolism | C5-Branched dibasic acid metabolism |
| Metabolism | Carbohydrate Metabolism | Glycolysis / Gluconeogenesis |
| Metabolism | Carbohydrate Metabolism | Pentose and glucuronate interconversions |
| Metabolism | Carbohydrate Metabolism | Pentose phosphate pathway |
| Metabolism | Metabolism of Cofactors and Vitamins | Folate biosynthesis |
| Metabolism | Metabolism of Cofactors and Vitamins | Nicotinate and nicotinamide metabolism |
| Metabolism | Metabolism of Cofactors and Vitamins | One carbon pool by folate |
| Metabolism | Metabolism of Cofactors and Vitamins | Riboflavin metabolism |
| Metabolism | Metabolism of Terpenoids and Polyketides | Carotenoid biosynthesis |
| Metabolism | Metabolism of Terpenoids and Polyketides | Tetracycline biosynthesis |
| Metabolism | Biosynthesis of Other Secondary Metabolites | beta-Lactam resistance |

## Table S3 – Alpha diversity indices for *Brachypodium distachyon* BD21experiment samples.

| **Host Environment** | **Time** | **N** | **Coverage (%)** | **S_obs_** | **Shannon^*^** | **ACE** |
| --- | --- | --- | --- | --- | --- | --- |
| *Brachypodium* | 1am | 9 | 95.8 ± 1.1 | 4386 ± 464 | 7.16 ± 0.14^A^ | 6375 ± 1762^A^ |
|  | 7am | 12 | 95.5 ± 1.5 | 4522 ± 652 | 7.22 ± 0.12^A^ | 7178 ± 2453^A^ |
|  | 1pm | 9 | 94.0 ± 0.7 | 5066 ± 314 | 7.16 ± 0.24^A^ | 9736 ± 1545^B^ |
|  | 7pm | 9 | 94.8 ± 1.4 | 4797 ± 545 | 7.23 ± 0.08^A^ | 8313 ± 2495^A,B^ |
| Soil | 1am | 9 | 96.9 ± 0.6 | 3257 ± 323 | 6.67 ± 0.17^A^ | 4669 ± 897^A^ |
|  | 7am | 12 | 96.6 ± 1.0 | 3369 ± 453 | 6.68 ± 0.19^A^ | 5465 ± 1692^A^ |
|  | 1pm | 9 | 96.3 ± 1.1 | 3462 ± 498 | 6.64 ± 0.21^A^ | 5988 ± 2619^A^ |
|  | 7pm | 9 | 96.4 ± 0.3 | 3522 ± 166 | 6.75 ± 0.12^A^ | 5326 ± 816^A^ |

S_obs_: OTUs observed; ACE: abundance-based coverage estimate of richness

^*^Times sharing the same superscript did not differ significantly by *post-hoc* test for a given host environment (*P* > 0.05).


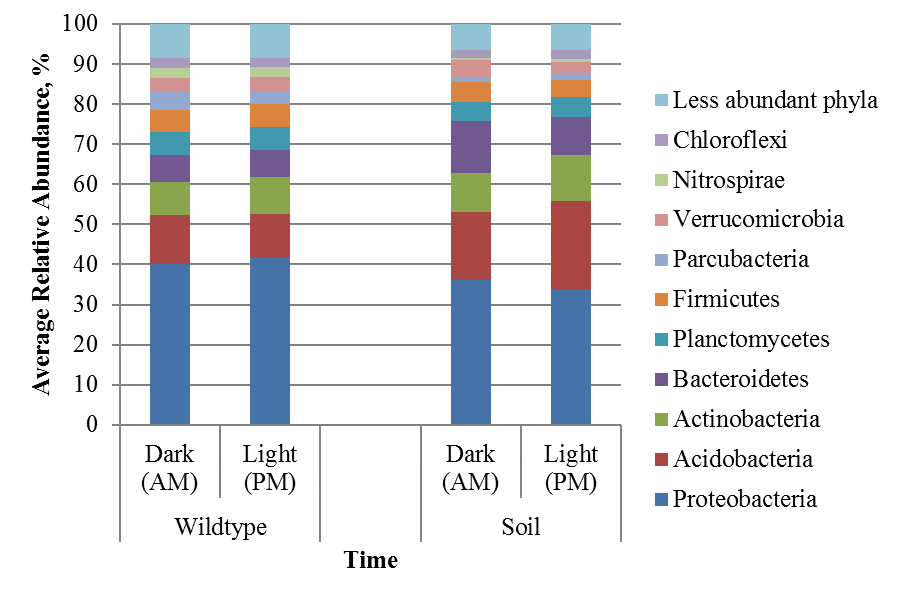


**Figure S1** – Distribution of abundant phyla among AM and PM samples in the rhizosphere of wildtype *Arabidopsis* and fallow soil.


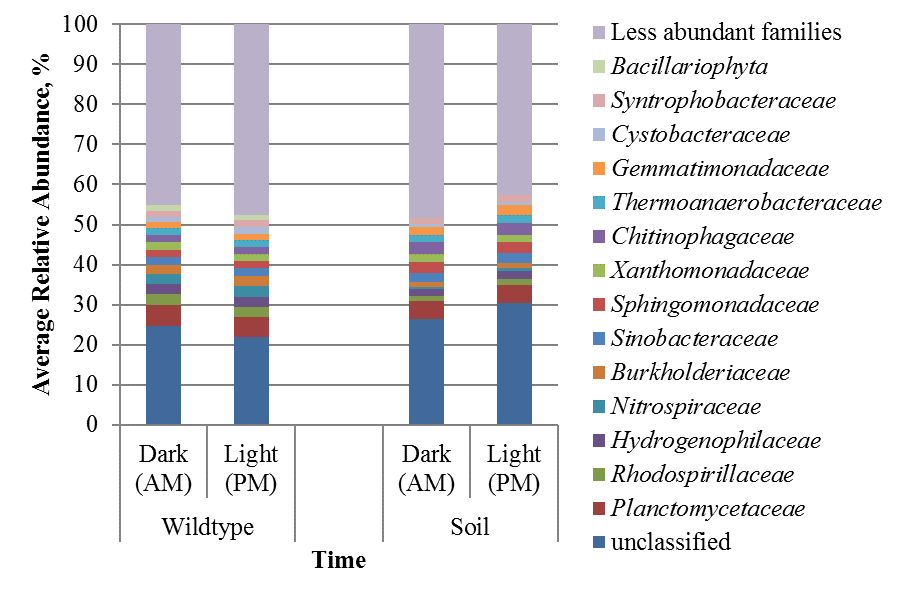


**Figure S2** – Distribution of abundant families among AM and PM samples in the rhizosphere of wildtype *Arabidopsis* and fallow soil.


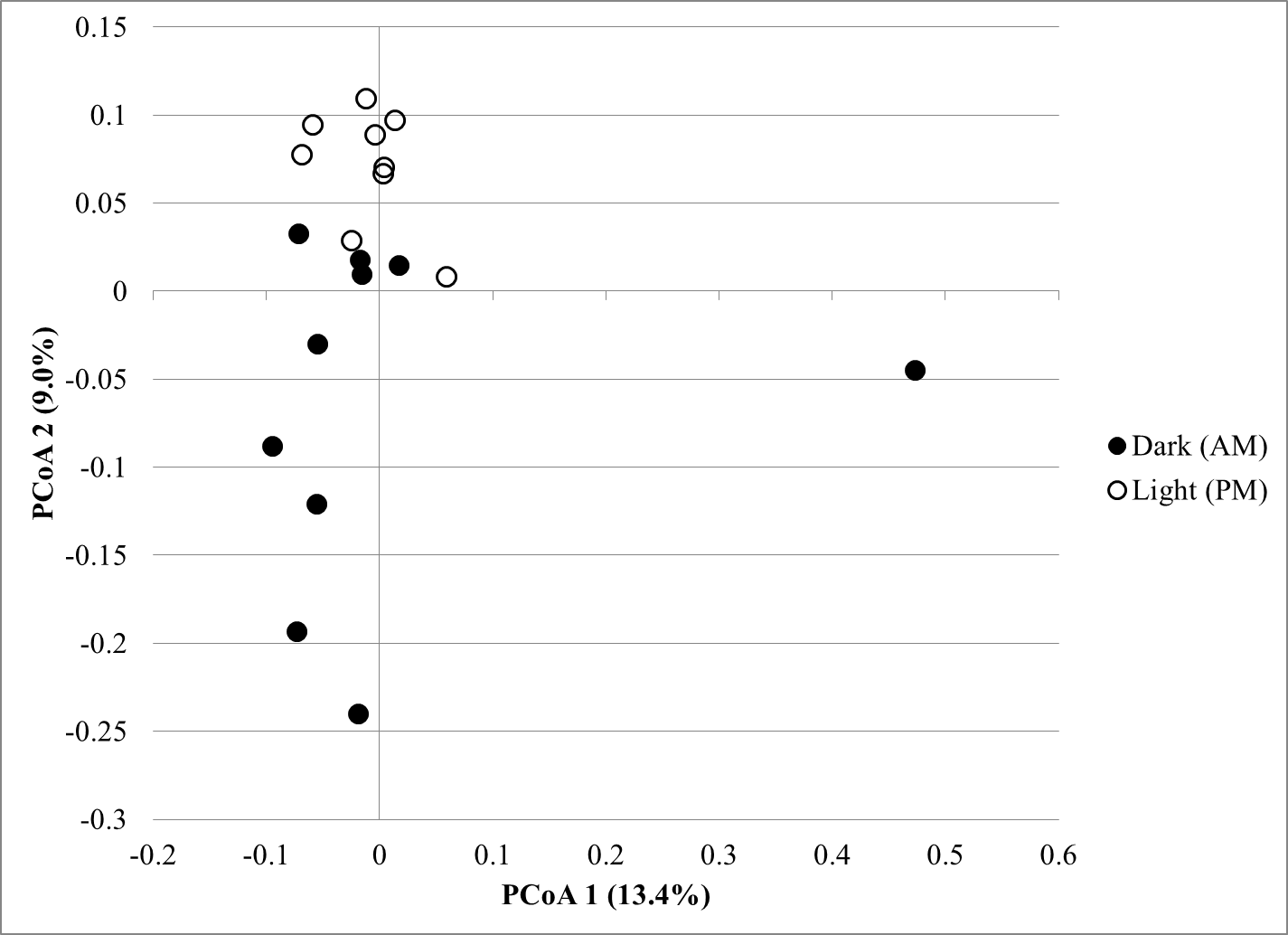

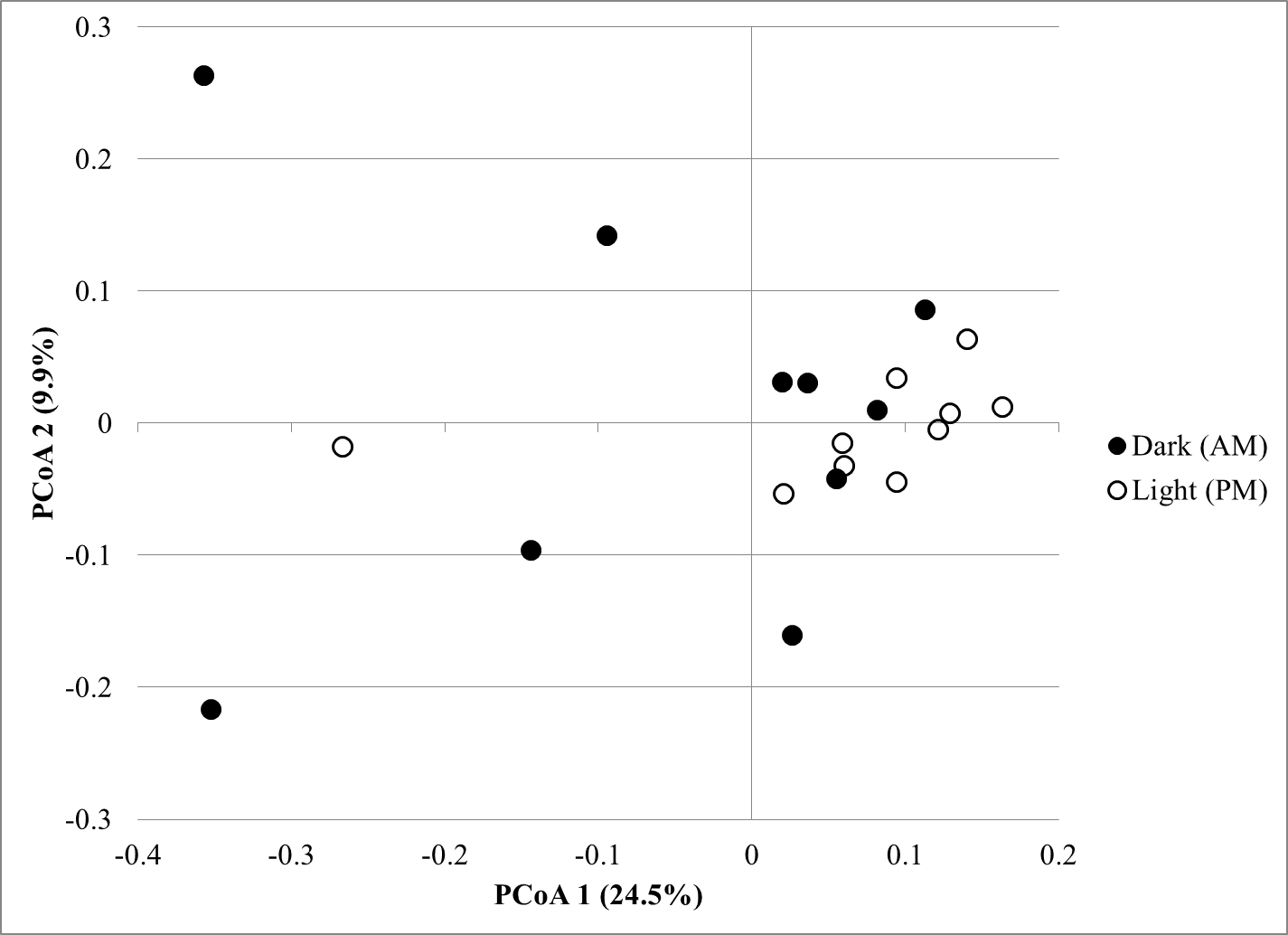


**B**

**A**

**Figure S3** – Principal coordinate analysis of Bray-Curtis dissimilarities among AM and PM bacterial communities characterized by 16S rRNA sequencing in A) the rhizosphere of *Arabidopsis* (r^2^ = 0.689) and B) fallow soil (r^2^ = 0.876). Points represent individual samples.

**Figure S4** – PCA plot of the relative abundance of the different organic compounds identified in each sample by water extraction (A) and van Krevelen diagram of elemental H/C (hydrogen –to-carbon) vs O/C (oxygen-to-carbon) ratios of the organic compounds extracted from the loading plot of the PCA plot (B).

B

A

**Figure S5** – 3D PCA plot of the relative abundance of the different compounds extracted by MeOH (A) and van Krevelen diagram of elemental H/C (hydrogen –to-carbon) vs O/C (oxygen-to-carbon) ratiosof the organic compounds extracted from the loading plot of the PCA plot (B))


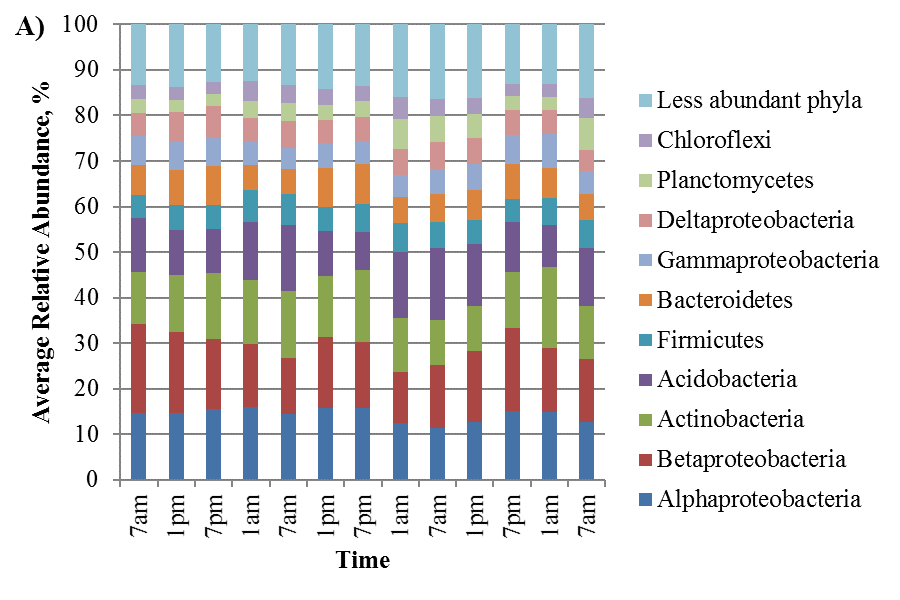

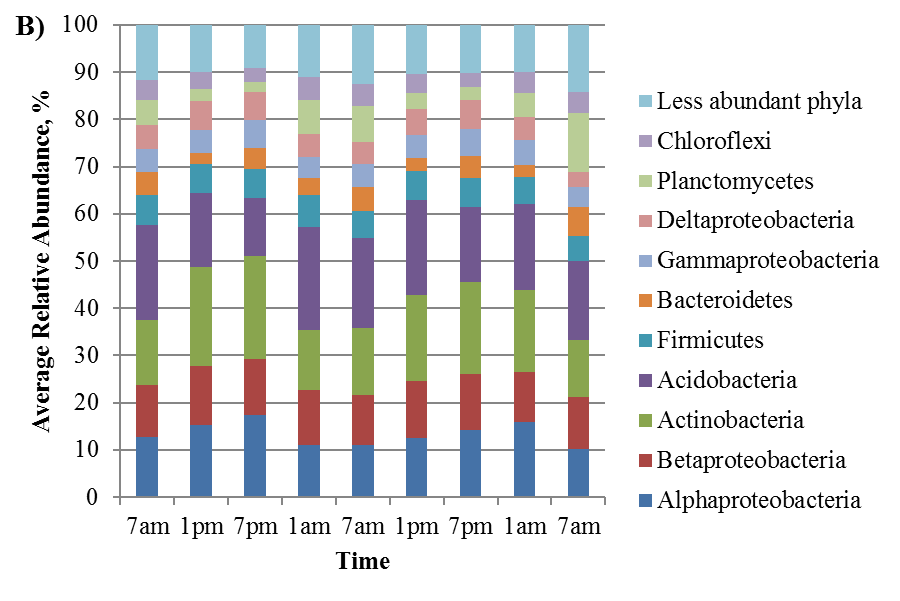


**Figure S6 –** Distributions of phyla in (A) *Arabidopsis* OX34 mutant rhizosphere and (B) fallow soil samples, omitting less abundant phyla. Percent abundances are cumulative. Sequence data from later time points for soil could not be obtained, and less abundant taxa are not shown.


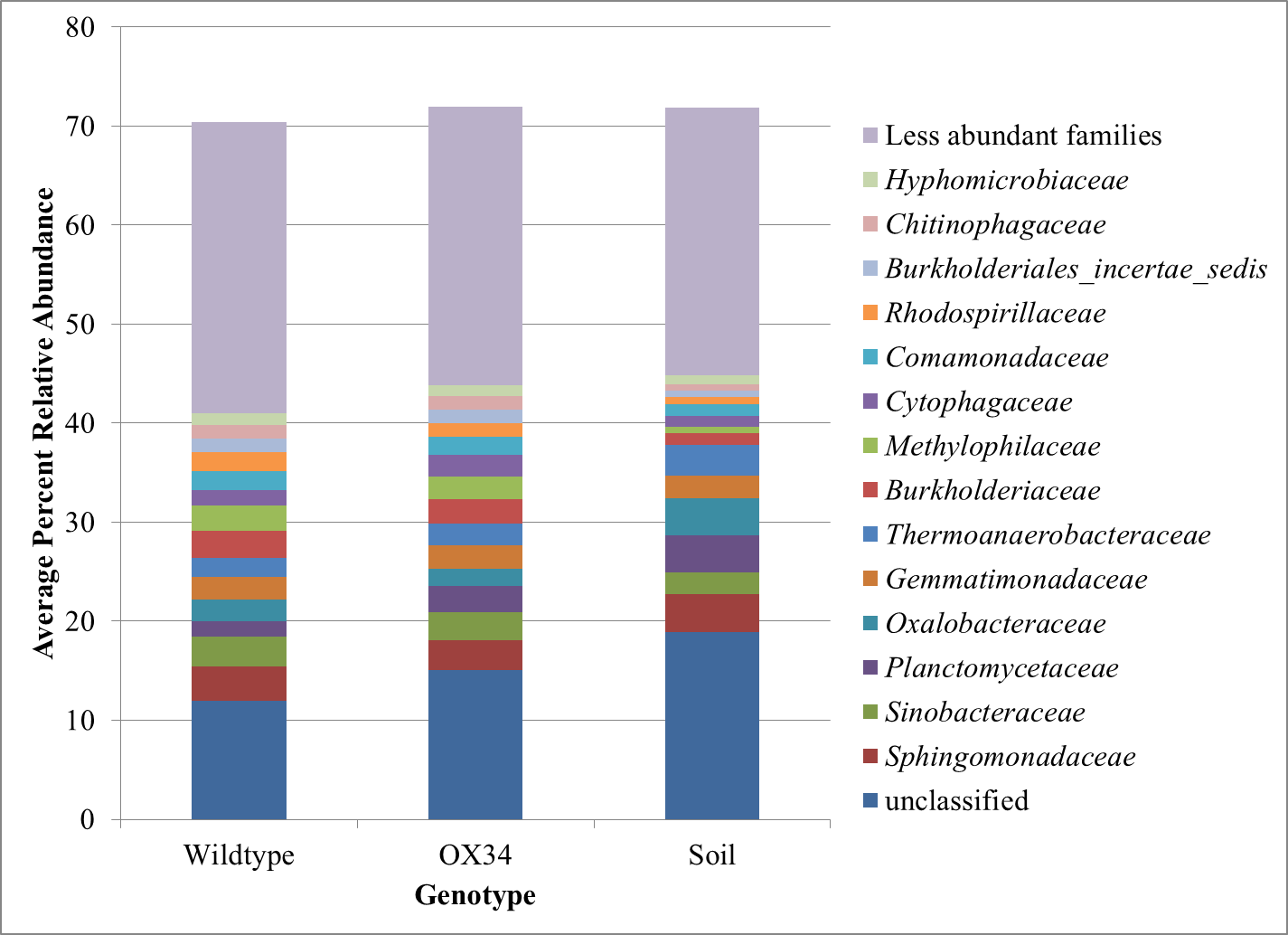


**Figure S7** – Family-level classification of OTUs that differed significantly among *Arabidopsis* rhizosphere genotypes and soil groups by Kruskal-Wallis test (*P* < 0.05).


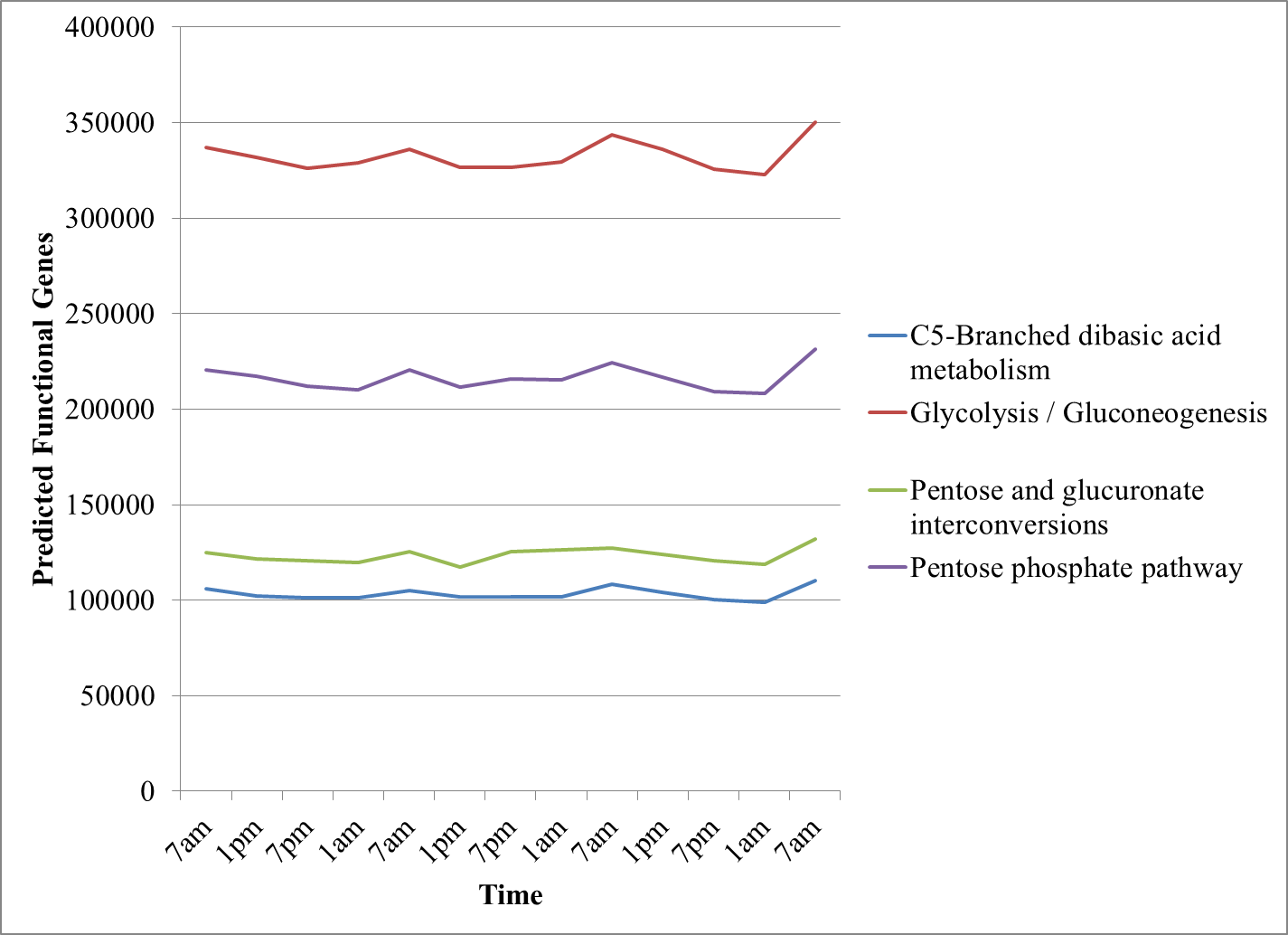
**Figure S8** **–** Inferred carbohydrate metabolism genes that showed significant cycling (*P* < 0.05) in abundance among wildtype *Arabidopsis* rhizosphere bacterial communities by the JTK algorithm.


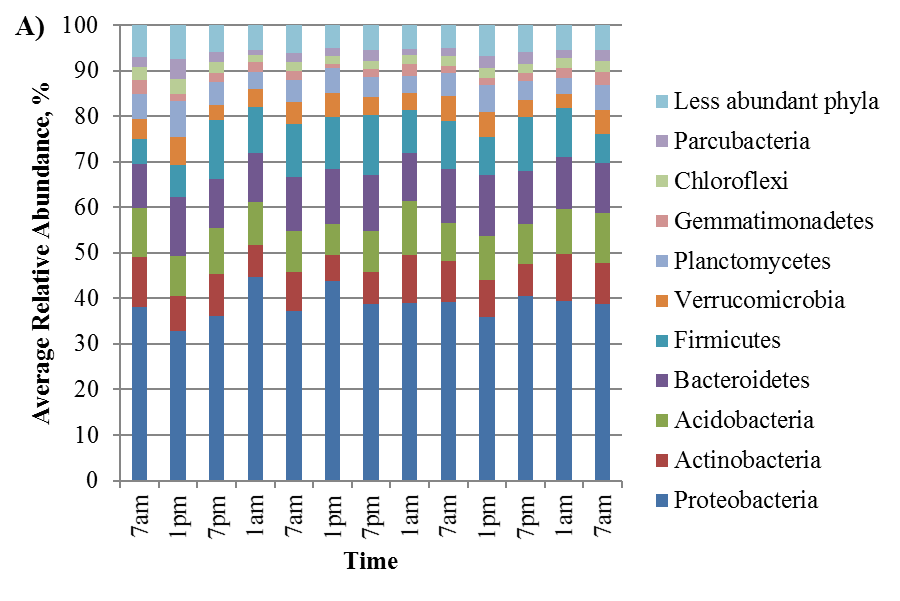

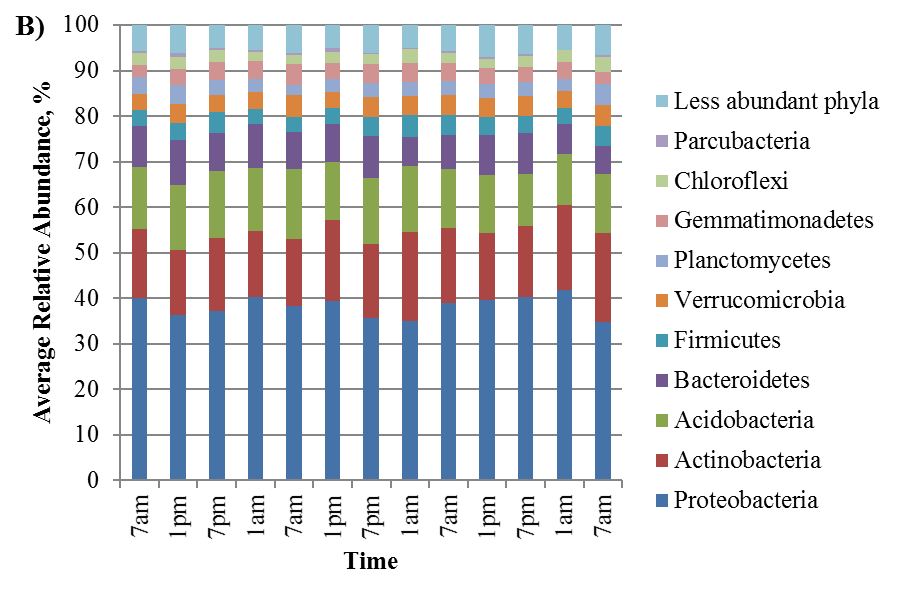


**Figure S9** – Distribution of phyla among samples from (A) the *Brachypodium* rhizosphere and (B) fallow soil. Forty-one less abundant phyla are not shown.

**
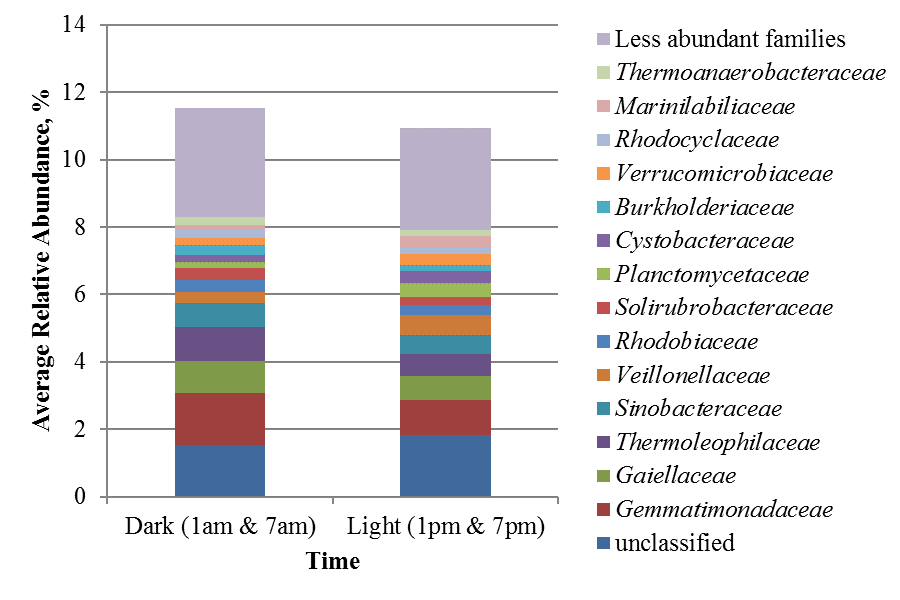
**

**Figure S10 –** Family-level classification of OTUs that differed significantly among light and dark periods by Kruskal-Wallis test (*P* < 0.05) in the *Brachypodium* rhizosphere.
